# A Facile Method Based on Faster R‑CNN for Cell Detection in Microfluidic Devices

**DOI:** 10.1021/acs.analchem.5c04533

**Published:** 2026-01-27

**Authors:** Guillaume Aubry, Yanjun Zhao, Erin Shappell, Jacob M. Wheelock, Hang Lu

**Affiliations:** † School of Chemical & Biomolecular Engineering, 1372 Georgia Institute of Technology, 311 Ferst Drive NW, Atlanta, Georgia 30332, United States; ‡ College of Arts and Sciences, 8025 Troy University, Troy, Alabama 36082, United States; § Interdisciplinary Program in Bioengineering, Georgia Institute of Technology, 311 Ferst Drive NW, Atlanta, Georgia 30332, United States; ∥ School of Electrical and Computer Engineering, Georgia Institute of Technology, 311 Ferst Drive NW, Atlanta, Georgia 30332, United States

## Abstract

Cell detection is ubiquitous in the analysis of microfluidic cell assays. In cell biology, immunology, oncology, and toxicology research, studying cellular response starts with identifying the cells on chip. The large amount of data generated in such assays requires automating image analysis. While multitudes of image processing tools exist, the microfluidic channel network and crowded cell environment make it difficult to identify and track cells by conventional image processing techniques. In contrast, machine learning-based techniques may overcome this challenge. Two important challenges in implementing these techniques are that it often requires tedious image labeling and coding expertise. Here, we present a facile method for cell detection in microfluidic arrays using Faster region-based convolutional neural network (R-CNN) that addresses both challenges. First, image labeling is fast and easy, because Faster R-CNN only needs bounding boxes as labels to generate training data. Second, we provide a ready-to-use model and a guide for training a Faster R-CNN model that does not require coding expertise. We demonstrate that Faster R-CNN does not need trade-offs between precision and user-friendliness: we created a model that detects cells with an average precision over 98% using a few hundred annotations, which takes less than half an hour. We show that shapes created by the microfluidic structure alone or its interplay with cells are not misidentified as cells. We show for the first time cell detection using Faster R-CNN in microfluidic chips; we envision that this approach will have a broad use in many on-chip fundamental biology and drug-discovery assays.

## Graphical abstract

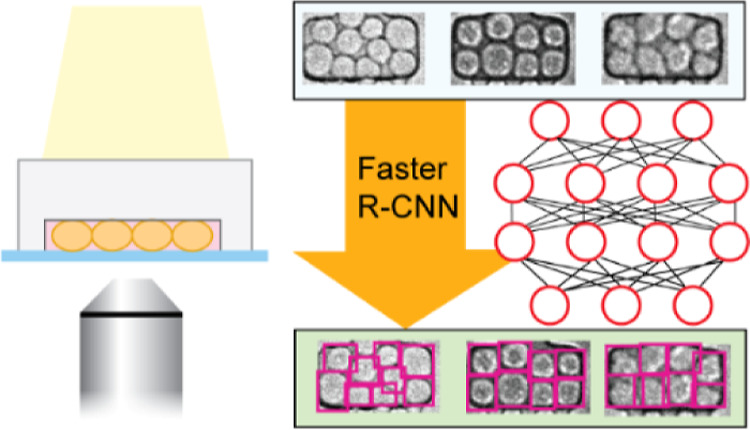

Microfluidic cell assays are widespread in research in cell biology,^1^ immunology,^2,3^ oncology,^4–6^ drug development,^7–9^ and personalized medicine.^10^ In these studies, image-based cell detection often is the crucial first step in the analysis, e.g., cell tracking, determining cell type and state, or quantifying cellular heterogeneity. However, processing the sheer volume of data generated by these assays presents a significant challenge. For example, longitudinal studies, drug-dosage assays, and patient-specific screens can easily produce tens of thousands of images. Manually analyzing such large data sets would require hundreds of hours of annotations and introduces the risk of user bias. To overcome these hurdles, there is a strong demand for automated image-processing methods that can expedite the analysis, streamline workflow, and eliminate human bias.

Numerous image-processing methods have been developed to automate cell detection;^11,12^ however, detecting single cells can be difficult in microfluidics. A variety of designs have been proposed to array cells on chip.^13,14^ The channel layout and cell clusters in bright-field imaging create complex scenes that can lead to false positive and false negative detections of cells. While fluorescence imaging can help in discriminating signals of dyed cells from background, the fluorescence signal of crowded cell environments results in one continuous object of uneven intensity that is difficult to split into individual cells. Therefore, there is still a need to develop automated image processing tools for detecting cells on chip.

While machine learning techniques have been developed to handle complex scenes in bright-field,^15^ they often require technical expertise and labor-intensive manual annotation. For example, CellProfiler^16^ requires the user to tune a series of parameters, and iLastik,^17^ an intensity-based clustering method, requires extensive annotation and is limited to targets with a clear intensity signature. Segmentation-based deep learning methods, such as U-Net,^18^ also share similar limitations: the training requires challenging cell contour annotation and the implementation requires coding proficiency.

In contrast, Faster R-CNN is a region-based convolutional neural network that returns the objects’ locations as rectangular regions of interest (ROI).^19^ Faster R-CNN may not return segmented objects but information about the presence and location of cells largely fulfills the needs of many cell-based assays that require cell enumeration. In return, training image annotation for Faster R-CNN is simplified to drawing boxes around individual cells, making the process time- and labor-efficient and demanding less expertise than segmentation-based CNN methods. While successful in detecting cells in conventional microscopy images, Faster R-CNN has not yet been demonstrated to work effectively in microfluidic environments.^20–33^ Because previous models lack training using images of cells in a microfluidic device, they tend to struggle to reliably distinguish foreground from background. Furthermore, previous Faster R-CNN studies may describe the structure of the neural network but lack accessible implementations, which excludes nonexpert users and prevents its broad use. Therefore, the potential of Faster R-CNN has yet to be demonstrated in microfluidic cell assays in a way that can be broadly and easily applied by nonexperts.

Here, we demonstrate that Faster R-CNN can be efficiently used to detect cells in microfluidic environments. To do so, we trained models with images of cells in different microfluidic layouts and taken under slightly different imaging conditions (i.e., with some differences in noise level and focal planes). Such training enables the model to reliably detect cells in complex scenes. We show that the models achieve high average precision over 98% for different objective magnifications and are robust to small variations in imaging conditions. We demonstrate its use in a cell killing assay upon chemical exposure, where we monitor survival rate in cell clusters with single-cell resolution. To set up and run the models, we used a publicly available and accessible implementation of Faster R-CNN.^34^ Importantly, this resource is readily usable for a large readership with no experience in programming; we also made available the model presented in this work that may directly fulfill the needs of a range of applications in microfluidic cell assays.

## Experimental Section

### Microfabrication

Microfluidic devices were fabricated using soft lithography.^35^ SU-8 2005 and 2010 photoresists (Kayaku AM) were used to create a multilayer master: 5 μm-high features for the back resistance channels, 15 μm-high features for the chambers, and 25 μm-high features for the rest of the channel network. The master was treated with an antiadhesive coating (tridecafluoro-1,1,2,2-tetrahydrooctyl-1-trichlorosilane, Sigma-Aldrich) under vacuum for 4 h. A 10:1 polydimethylsiloxane (PDMS, Dow Corning SYLGARD 184) to cross-linker ratio mixture was then cast on the master and left in the oven at 90 °C overnight. PDMS chunks were then cut off, access wells punched, and the PDMS slabs were irreversibly bonded on glass slides via oxygen plasma treatment (Plasma Wand, Plasma Etch Inc.).

### Cell Culture and Reagents

K-562 lymphoblast cell and T cell lines were cultured in RPMI-1640 media (ATCC) supplemented with 10% fetal bovine serum (FBS, VWR). HT1080 fibrosarcoma cells were cultured in DMEM medium (ATCC) supplemented with 10% fetal bovine serum (FBS, VWR). The culture flasks were stored in an incubator at 37 °C and 5% CO_2_. Cells were passaged every 2 days at 10^5^ cells/mL. Reagents used for the assay include phosphate-buffered saline (PBS, Sigma) for priming the devices, fluorescent reporter (CellEvent Caspase-3/7 Green Flow Cytometry Assay Kit) to monitor cell apoptosis, and hydrogen peroxide H_2_O_2_ (Sigma) as toxic reagent.

### Microscopy

Images of microfluidic arrays loaded with cells were acquired on a confocal microscope (Nikon W1 spinning disc confocal) equipped with a sCMOS camera (Hamamatsu ORCA-Fusion Gen-III sCMOS). Different objectives were used: 10x, and 20x objectives.

### Image Preprocessing

Whole-array images were automatically cropped into series of individual-chamber images via a separate Faster R-CNN model (using same EZ-FRCNN package [34] from that for cell detection—see next section). Training of the chamber-detection model was performed with 156 chamber annotations in one whole-array image and run over 200 epochs. The overall loss plateaued at ∼0.15. The chamber-detection model was tested on four new images. Oversized inferences and inferences on the edges (corresponding to chambers partially out of the camera’s field of view) were filtered out. The model achieved high performances with 100% precision and 96% recall.

### Faster R-CNN Training and Performance Evaluation

All models were trained and run using the EZ-FRCNN package.^34^ EZ-FRCNN is a locally hosted application and provides a GUI and a Jupyter notebook to implement Faster R-CNNs. The network structure consists of a ResNet-50 backbone for feature extraction, enhanced by a feature pyramid network (FPN) to detect objects at multiple scales. A region proposal network (RPN) generates candidate regions, which are classified and refined by the detection head to identify and localize objects. An existing Faster R-CNN model pretrained on the COCO image data set is used as a starting point. The model is then further trained using images of microfluidic chambers filled with cells. Typically, training takes 30–60 min on CPU (Intel­(F) Xeon CPU E5-2680 v4 @2.40 GHz). No GPU was used for training a model or inferring images.

A threshold of 0.9 was used for the confidence score to select for relevant inferences. The models’ performances were evaluated by calculating the Precision = TP/(TP + FP) and Recall = TP/(TP + FN), where TP, FP, and FN stand for true positive, false positive, and false negative, respectively. TP, FN, and FP detections were determined using the criterion intersection over union IoU = (area of intersection of GT and P)/(area of union of GT and P), where GT is the ground truth box area defined by the annotator and P is the box area inferred by the model. A threshold of 0.5 was used for the IoU to distinguish TP and FP events unless otherwise specified. Inference speed was measured as the average processing time when inferring cells in a batch of 30 images.

When studying the performances as a function of training size, five separate models were trained and evaluated for each training set size. The images composing the training set were pooled randomly from a large deck of manually annotated images. All models were evaluated against the same testing set composed of images that were not part of the image pool used for the training sets.

### Cell Detection Using Other Methods and Performance Evaluation

Two additional methods, one traditional and one based on machine learning, were tested to compare with the performances of Faster R-CNN. The traditional method implements the circular Hough transform to detect circles in the images. We used the MATLAB built-in function “imfindcircles” to process the images and a size filter to select circles with radius comprised between 5 and 100 pixels. The machine learning based method used first iLastik (https://www.ilastik.org/) to segment the images and then MATLAB to apply a size filter and a shape filter. The images used in iLastik for training are the same as the 40-image training set used for the Faster R-CNN method. Precision and recall were calculated the same way as described in the section on Faster R-CNN. Bounding boxes corresponding to the circles detected in the traditional method and the areas segmented via iLastik were used to determine the IoUs with the ground truth.

### Cell Killing Assay

All materials, devices, tubing, pipettes tips, clamps were autoclaved at 110 °C for 30 min. Reagents were maintained sterile using 0.20 μm filters (Corning Inc.). All further operations were performed in a sterile environment under a biohood. Devices were primed and degassed with PBS. K562 cells were resuspended in RPMI-1640 media at 10^5^ cells/mL and loaded in the microfluidic arrays. Experimental conditions were determined based on the work of Li et al.^36^ A 1 mM H_2_O_2_ solution containing the caspase-3/7 fluorescent reporter at 3 mM was perfused through one device via gravity flow over the entire course of the assay. The device was imaged every hour for 8 h. A higher H_2_O_2_ concentration than in^36^ was used to compensate for dilution effect coming from the dead volume in the pipet reservoir at the device inlet. One culture medium control solution was perfused in a second device and imaged at the beginning of the assay and at the end 8 h later. Both bright-field and fluorescence images were taken on a spinning disc confocal microscope at 10× magnification (Nikon W1 spinning disc confocal).

Note that change of cell morphology may happen during apoptosis. To maintain efficient cell detection through the assay, the assay duration was limited to 8 h: enough to observe the activation of apoptosis signaling pathway with the caspase 3/7 reporter but not too long to limit both cell degradation and cell division. In addition, by design of the array, the cells are trapped in chambers and cannot escape. Therefore, the total cell number in the array remains constant over time. Based on this assumption, cell detection was performed at t0 by selecting all inferences with a confidence score of 0.9 or higher. At later time points, the confidence score was lowered such that it would match the N_total determined at t0. This presumably catches the dying cells. The number of dead cells were then characterized from the caspase fluorescence.

### Data Analysis of the Cell Killing Assay

Time-lapse series of the whole-array images were split into time-lapse series of individual chambers. Cell detection was performed on the bright-field images via the Faster R-CNN model. Cells’ live/dead status was determined by analyzing the level of green fluorescence within the detection box of the inferences. The live vs dead threshold was selected using the histogram of fluorescence cell signal at *t* = 8 h post H_2_O_2_ exposure. The histogram displays a bimodal distribution and the local minimum between the two peaks was chosen as the threshold.

## Results and Discussion

### An Easy-to-Implement Method for Cell Detection

To make the analysis pipeline accessible to a large public regardless of programming experience, one needs a method that is fast and easy to implement. To address this challenge, we opted for a Faster R-CNN implementation that uses PyTorch and Docker.^34^ Figure 1 highlights the minimal user input through each step of the process.

**Figure 1:**
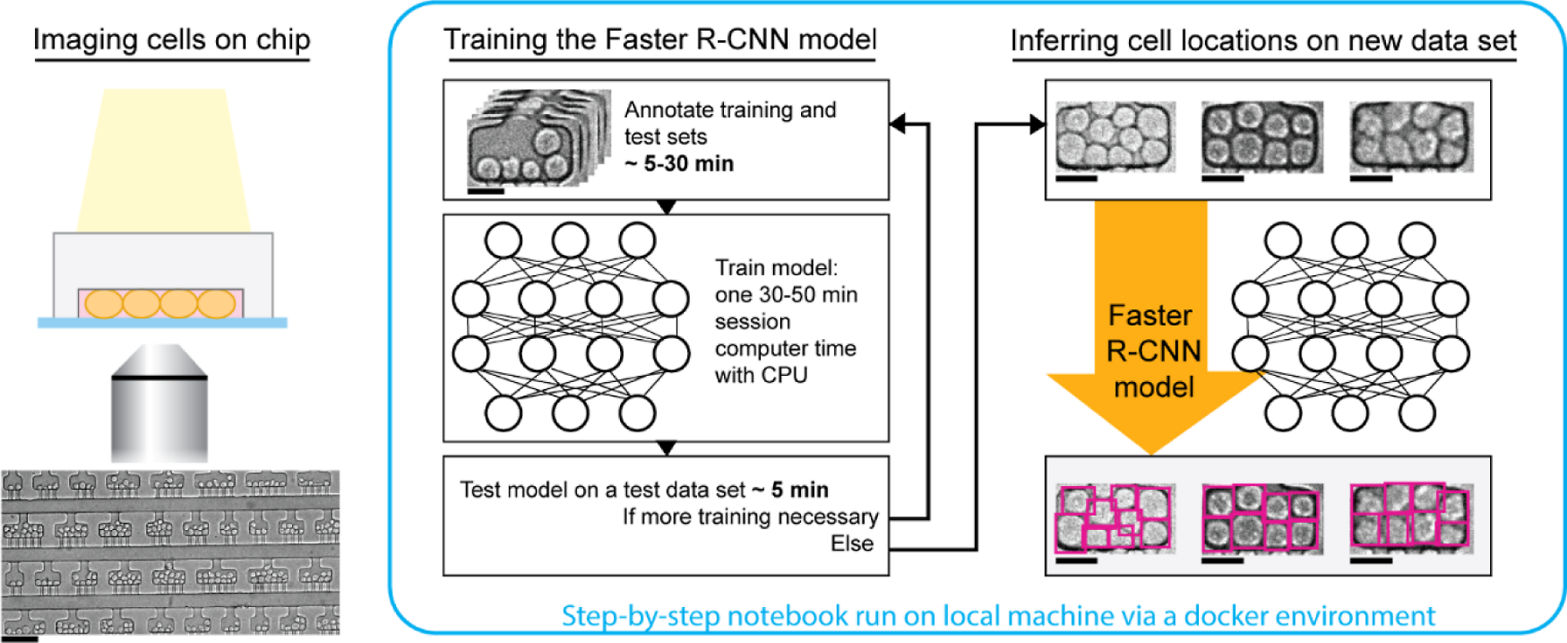
A simple implementation scheme based on Faster R-CNN to detect cells in microfluidic devices. (1) Take images of cells in microfluidic devices. We recommend adjusting focus to optimize contrast of bright-field images. (2) Train a Faster R-CNN model in less than 2 h, with the time requiring human intervention less than 40 min. (3) Use the model on new images to detect cells. Scalebars: 100 μm for the zoom-out view of the array and 25 μm for the zoomed-in view of individual chambers.

The first step is the annotation of training images, which is fast and easy. By leveraging a model pretrained on the broad COCO data set, only a small number of images is necessary for model tuning to the user’s specific needs. Annotation itself is also simple and fast, since it only requires drawing boxes around the cells. This step can take as little as a few minutes, depending on the application.

The second step is training the neural network, which is also fast. Executing a typical training length of 200 epochs takes ∼30–50 min CPU-time. The training does not require any work from the user; the model goes through a series of optimizations until the user stops the training. After checking the model performances, one can further tune the model or use it to analyze new data sets.

Once the model is trained and tested, inferring cell position on new images is straightforward. Experimental images are fed to the model and it returns the coordinates of the detection boxes in a spreadsheet. With a CPU, the process takes on average 2.5 s per 200 × 100 pixel image and can work in batches, which makes it more time efficient for the user. Using a GPU may considerably accelerate the processing time.

### High Performance for Diverse Imaging Conditions

To extract reliable information from on-chip cell assays, one needs a method that can deliver high performance in microfluidic environments. Detecting cells on chip is not straightforward because of the PDMS structures, varying imaging conditions, and dense cell clusters. We studied and addressed each aspect via the case study of lymphoblast cells in microfluidic chambers imaged with a 10× objective.

First, the PDMS-water refractive index mismatch makes the channel contour highly visible, which may mislead the neural network. To solve this challenge, we included images of four different microfluidic chambers in the training set. The chambers vary in size and number of outlet channels (Figure 2a). The model learns properties of the cells that are invariant from the diverse device designs; therefore, such training will allow the model to detect cells in microfluidic background. We completed the training of the model using 60 images of single microfluidic chambers, which is very easy to annotate. The training set contains 410 annotations of cells distributed in the different chamber types (Figure S1). The resulting Faster R-CNN model (File S1) performs very well at detecting cells in the different chambers. Figure 2a shows representative microscopy images, which were not part of the training set, and the green rectangles indicate the inferences obtained with the Faster R-CNN model. The trained model accurately identifies the cells and the microfluidic channels do not generate false positives.

**Figure 2:**
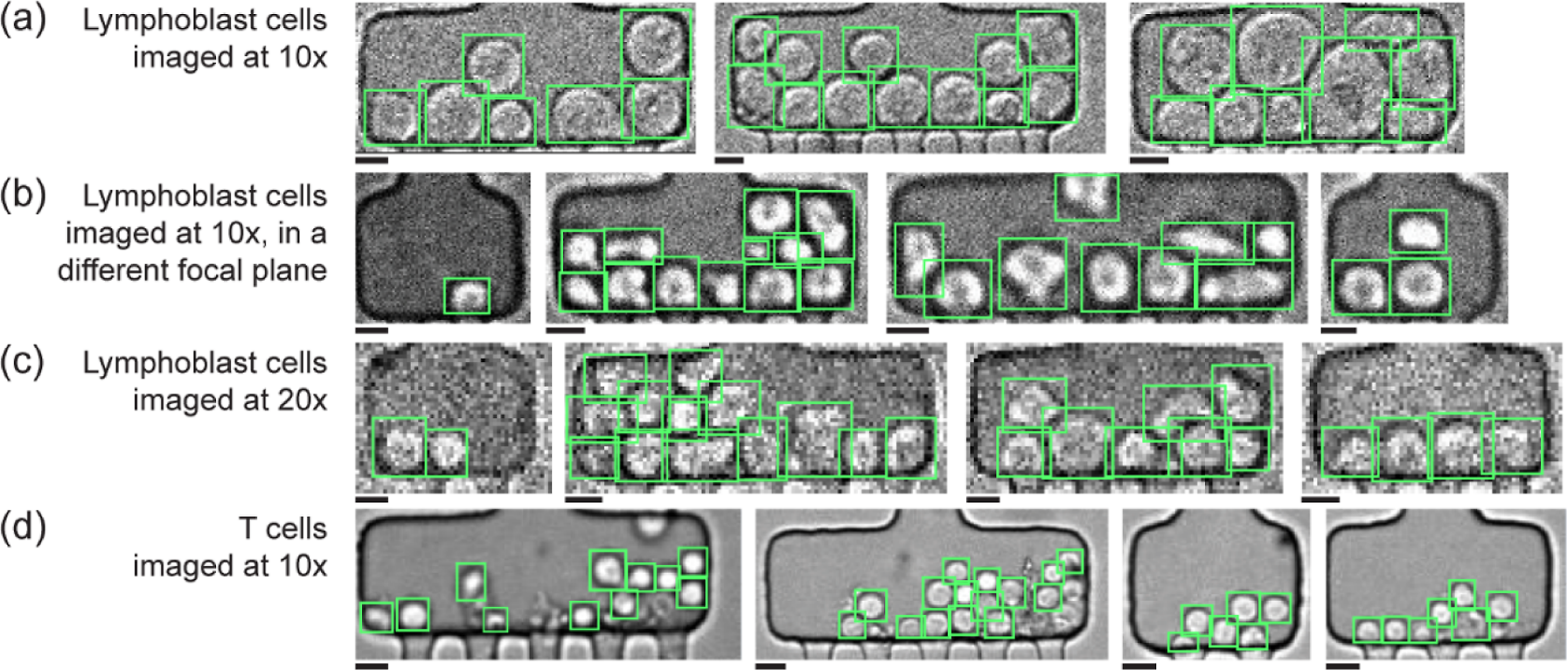
Representative results of cell detection in microfluidic chambers. Green rectangles locate the inferences generated by the Faster R-CNN model. (a) Inferences of lymphoblast cells imaged at 10× in one focal plane and (b) inferences of lymphoblast cells imaged at 10× in a different focal plane. (c) Inferences of lymphoblast cells imaged at 20×. (d) Inferences of T cells imaged at 10×. Scalebars: 10 μm.

Second, cell detection becomes even more challenging as imaging conditions may change from imaging session to imaging session, and from device to device. A change in focus plane, variations in the illumination intensity, and different camera settings can have a significant impact on the image quality. To address this issue, we used images taken under slightly different imaging conditions for training. For example, collected images were taken within 10 μm from focal plane and the relative standard deviation of pixel intensities per image varied between 15% and 27%. Then, to test the robustness of the model to different imaging conditions, we analyzed images of the same cell line imaged at different focal planes and noise levels (Figure 2b). The contours of the cells appear blurrier, and the cell images overall contain less detail. The neural network successfully detects the cells in the chambers across these varying conditions.

Third, cells trapped in microfluidic chambers may lead to a variety of configurations from single cells to large clusters. Densely packed clusters are particularly challenging to resolve because the shape of the cells changes and gaps between cells may be mistakenly inferred as cells. To address this issue, we included images of microfluidic chambers containing 1 to 15 cells each in the training set. Figure 2a,b show that cells are reliably detected even in crowded environments, the bounding boxes tightly frame the cell contours, and no gaps between cells or cells and channel walls are mistaken as cells.

To further characterize the performances of the model, we quantitatively evaluated the model’s precision on a test set of new images. The testing set contains 140 images of cells in a chamber (total of over 900 cells) and includes a variety of chamber sizes and numbers of cells trapped in the chambers. The average precision (AP) of the model’s performance was over 98%.

Next, to showcase the broad use of the method across imaging setups, we performed cell detection for a different microscope objective without having to retrain the model. We acquired images of 250 lymphoblast cells at 20× objective magnification. At higher magnification, the cytoplasm shows more texture than in the 10× images. Despite this difference, the Faster R-CNN model detects the cells very well (Figure 2c) with an AP of 93% for a confidence score threshold of 0.9. This indicates a strong model confidence that the detected objects belong to the targeted class. An IoU threshold of 0.5 was used, a commonly accepted cutoff in object detection to define a valid detection. These results show that the model trained under one imaging set up may be valuable for different microscopy settings, which offers greater flexibility and saves time.

To further demonstrate the generalizability of the method, we also analyzed images of a different cell line without retraining the model. T cells are smaller; they are typically ∼10 μm in diameter vs ∼15 μm for the lymphoblast cell line. The model detects the T cells (Figure 2d) with a precision of over 80% for a confidence score of 0.8 and IoU of 0.5. If higher precision is desired, one may tune the model further by training with images of the new cell line. Given the ease-of-use of the platform (Figure 1), annotating and retraining the model to tune the model would likely lead to higher performances within a couple of hours.

Models specific to microfluidic environments are necessary to achieve high performances. To demonstrate that existing Faster R-CNN models are not adequate to detect cells on chip, we developed a model equivalent in terms of environment to those published in the literature [20–33]. We used cells in wells; similar results can be obtained using cells on glass slides. We show that models trained on images without microfluidic features fail at detecting cells in microfluidic devices. (Figure S2). For training, we used images of the same cell line taken at the same magnification and similar image quality (Figure S2a).

We tested the performances of the model on images of cells on chip, using the same testing data set used previously. The model performed very poorly with a recall of 34% for a confidence score of 0.9 and IoU of 0.5 (Figure S2b). Lowering the threshold for the confidence score to 0.1 (and keeping the same IoU) did not improve the performances: many cells are still missed and false positives are generated (Figure S2c). In contrast, the model trained on images of cells in microfluidic images performs very well (Figure S2d). Furthermore, because imaging conditions may vary greatly for different experiments, microscopy set-ups, and laboratories, having the ability to easily tune a model enables greater flexibility to optimize the model performances to a given assay. This highlights the benefit of having a specific model for microfluidic environments.

### Model Performance Requires Minimal Training Data and is Robust Against Annotator Bias

Obtaining the best performances of the Faster R-CNN requires a good understanding of the impact of the user’s personal bias. While the Faster R-CNN end-to-end structure minimizes user input, the user still plays an essential role during the training phase. The model will be as good as the training data is representative, the labels match ground truth, and the total loss is minimized. The user has a direct impact on these three aspects through selection of the series of images of the training set, annotation of the images, and the selection of the training duration. To provide some guidance on which parameters are sensitive and what typical range to use, we investigated the impact of the size of the training set and annotation on the model performance.

First, we asked what the minimal number of images is required for a model to perform well. To estimate the impact of the size of the training set on the Faster R-CNN performances, we compared the performances of models trained on 1, 40, and 400 cell annotations distributed among 1, 4, and 60 images, respectively. For each training size, five models were trained on five different training sets and evaluated. The training was terminated once the number of iterations was over 200 epochs and the total losses fell below 0.15, ensuring that the total losses plateaued at a level well-below 1, which is considered a significant training. Figure 3a shows the average precision of the models tested on the same image test set. We observe an increase of the precision with the size of the training set, in particular from 1 to 40 cell annotations. Above the 40-annotation training set, the precision plateaus.

**Figure 3:**
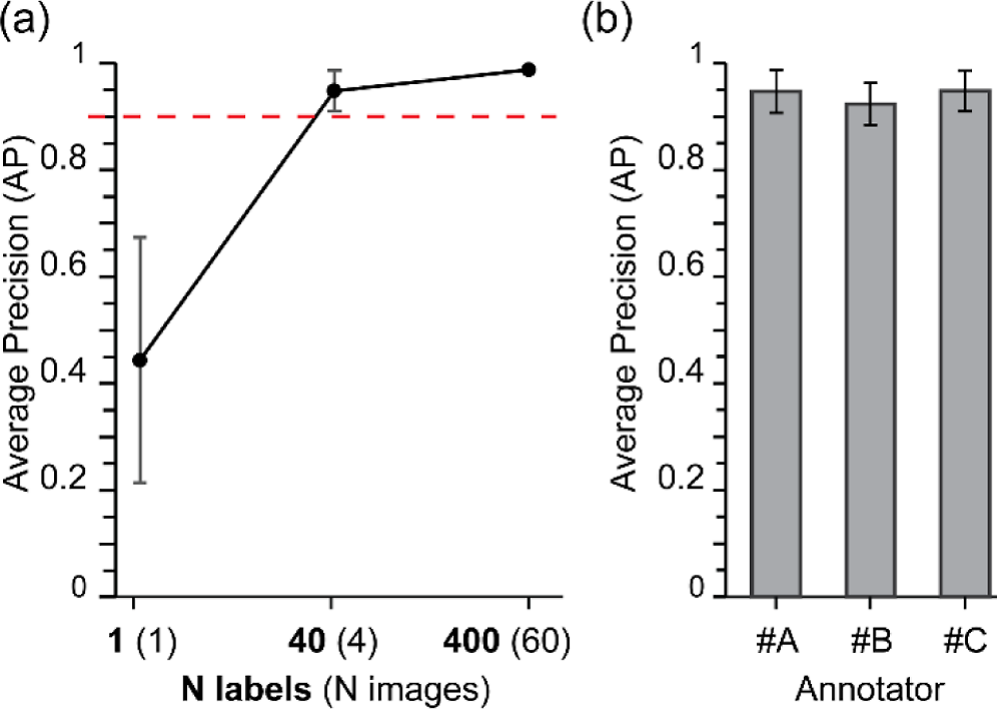
Impact of the size of training set and annotator variability on the Faster R-CNN performances. (a) Increase of average precision with the number of cell annotations (N_labels) in the training set. N_images indicates the total number of images for each training set. Dashed red line highlights an AP threshold of 0.9, above which the Faster R-CNN performances are considered highly satisfying. (b) Homogenous high average precision of models trained on data sets annotated by three different individuals.

This result illustrates the importance of avoiding a low number of annotations: when the training set lacks diversity, this leads to an under-trained subperforming neural network. We show here that 40 annotations lead to a high performing model. A fewer number of annotations might even be sufficient; however, the gain in time and effort would not be meaningful as annotating 10 cells instread of 40 cells would shorten the annotation time by 1 min. On the other hand, training on data set of hundreds of images does not hurt the NN performances but may be unnecessary. Annotating larger training sets requires more manual labor and time, and the gain in precision between the 400- and 40-cell-annotation-based models is not significant. Another parameter is the number of individual images that adds diversity in the training, for example via different backgrounds, lightning, and cell distribution. Interestingly, we show here that limited information about the background allows for high performances as the model reaches 95% AP using four images versus 98% using 60 images.

For a different microfluidic cell assay, one would expect a comparable number of images as long as the diversity in image content and image quality remain similar. When imaging cells in suspension in a different microfluidic array, our current model may serve as a good starting point, and one may optimize training with only a few new images. With cells of different morphology, such as adherent cells, or cells in very different microfluidic channel layout, one would need to train a new model on tens of images and assess performances. We tested a model trained on HT1080 adherent cells and obtained promising results (Figure S3). Overall, these findings highlight the interest of balancing training set size with annotation effort, as well-chosen data sets can achieve near-optimal performance with significantly reduced manual input.

Second, to make the analysis as reliable as possible requires the cell detection to be independent of the annotator. In principle, the results should not be annotator specific because the ground truth does not depend on the annotator. However, one cannot exclude human errors and implicit bias due to differences in human perception. To test the robustness of the R-CNN to the annotation process, we asked three individuals A, B, and C to annotate the same 200-cell image set and trained five 40-label-based models for each annotator. We compared the performances of the models (Figure 3b). The performances are very close with average precisions of 94%, 92%, and 95% for annotators A, B, and C respectively, leading to a standard deviation of ∼1.5%, which is negligible for general use. Note that the three annotators only annotated the training set and not the testing set. The testing set was annotated by a fourth person and trusted to be the ground truth. A benefit of having the process not annotator-specific is that one can use multiple annotators to facilitate the annotation. One can, in this way, speed up the process, make the annotation task easier, and target more demanding tasks with larger training sets.

### Faster R-CNN Outperforms a Conventional Method and a Popular ML-Based Method

To demonstrate that Faster R-CNN can outperform some popular, easy-to-implement image processing techniques, we compared the performances of our model with a conventional circle detection method and a machine learning-based iLastik method. The circle detection method finds circles using a circular Hough transform (MATLAB “imfindcircles” function). This algorithm is popular because of its robustness in the presence of noise, occlusion and varying illumination. The machine learning iLastik software (https://www.ilastik.org/) is a free software that uses pixel intensity clustering to segment objects. iLastik is also a popular analysis tool because of its user-friendly interface and low number of parameters to enter.

We show that Faster R-CNN performs better for cell detection tasks than both the circle detection and iLastik methods. Figure 4a shows a representative image with the inferences found by the Faster R-CNN (green rectangles in -i-), the objects detected by the circle detection algorithm (orange circles in -ii-), and the instances segmented in iLastik and then filtered by size and shape (orange shaded areas in -iii-). Faster R-CNN accurately detects all the cells in the chamber. On the other hand, the circle detection method generates multiple errors. Cells whose shapes are less circular often lead to inaccurate partial detections, and in some instances several circles detected for the same cell. The iLastik based method also presents multiple errors: the absence of a clear intensity signature distinguishing the cells from the background leads to multiple false negatives (see the cells pointed by white arrows of Figure 4a­(-iii-)) and false positives (see the microfluidic channels at the bottom of Figure 4a­(-iii-)).

**Figure 4:**
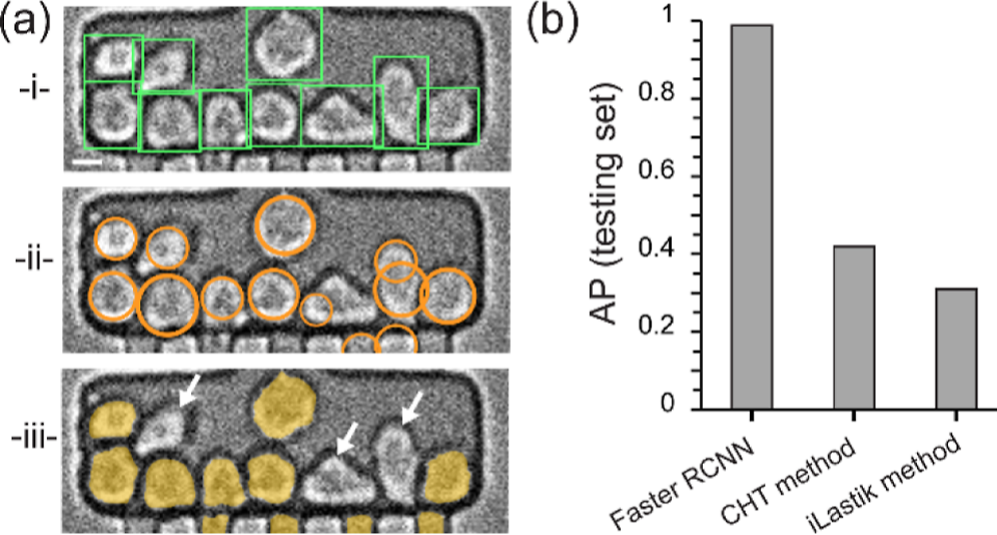
Comparison of Faster R-CNN performances with a conventional circle-detection method and a machine learning-based iLastik method. (a) Representative cell detection for the same image with Faster R-CNN (green rectangles in -i-), the circle detection method (orange circles in -ii-), and iLastik method (shaded orange areas in -iii-). White arrows point at false negatives. Scalebar: 10 μm. (b) Quantification of the three methods using the same image testing set.

A quantitative comparison of the performances of Faster R-CNN with the other methods confirms that Faster R-CNN delivers overall better average precision (Figure 4b). The circle detection method has a 40% precision and the iLastik based method points at 30%. Both are largely below Faster R-CNN, which precision is over 95%. These results demonstrate that Faster R-CNN does not trade off precision for user-friendliness.

We acknowledge that segmentation-based NN models can likely process the same images given proper training. However, such methods often require more know-how in setting up the software and therefore their access to non-engineer laboratories may be limited. This goes beyond time and expertise required for annotating images. The expertise in setting up a program is also a barrier that can prevent first-time users to access state-of-the-art AI techniques. Some efforts have been made to render segmentation-based NN more accessible and we tried CellPose-SAM (https://www.cellpose.org/), an application of CellPose that runs online and does not require setting up a program or training a model. The inferring time was very long (∼70 s per image) and the results were suboptimal (Figure S4). In contrast, EZ-FRCNN is very easy to set up (https://ezfrcnn.com) and we demonstrated that minimal training delivers high precision (Figure 3a). Therefore, our method provides an efficient way of reliably detecting cells on chip.

### Application to Quantifying Cell Survival Upon Chemical Exposure

To showcase the application of Faster R-CNN in a microfluidic cell assay, we performed a simple cell killing assay and used the Faster R-CNN model to determine the percentage of cell survival through time. Cell killing assays are essential in toxicology, pharmaceutic industry, and precision medicine. They are used to establish critical dosage, establish drug efficiency, and customize patient treatments. As a proof-of-application, we loaded lymphoblast cells in a microfluidic array and perfused the device with an H_2_O_2_ solution at 1 mM (Figure 5a). We also ran a negative control on chip, where the cells were perfused with culture medium. We monitored the cell response through time by taking bright-field and fluorescence images every 2 h for 8 h. The fluorescence images capture the signal of a green fluorescent reporter for apoptosis. Figure 5b shows a clear response of cells perfused with the H_2_O_2_ solution.

**Figure 5:**
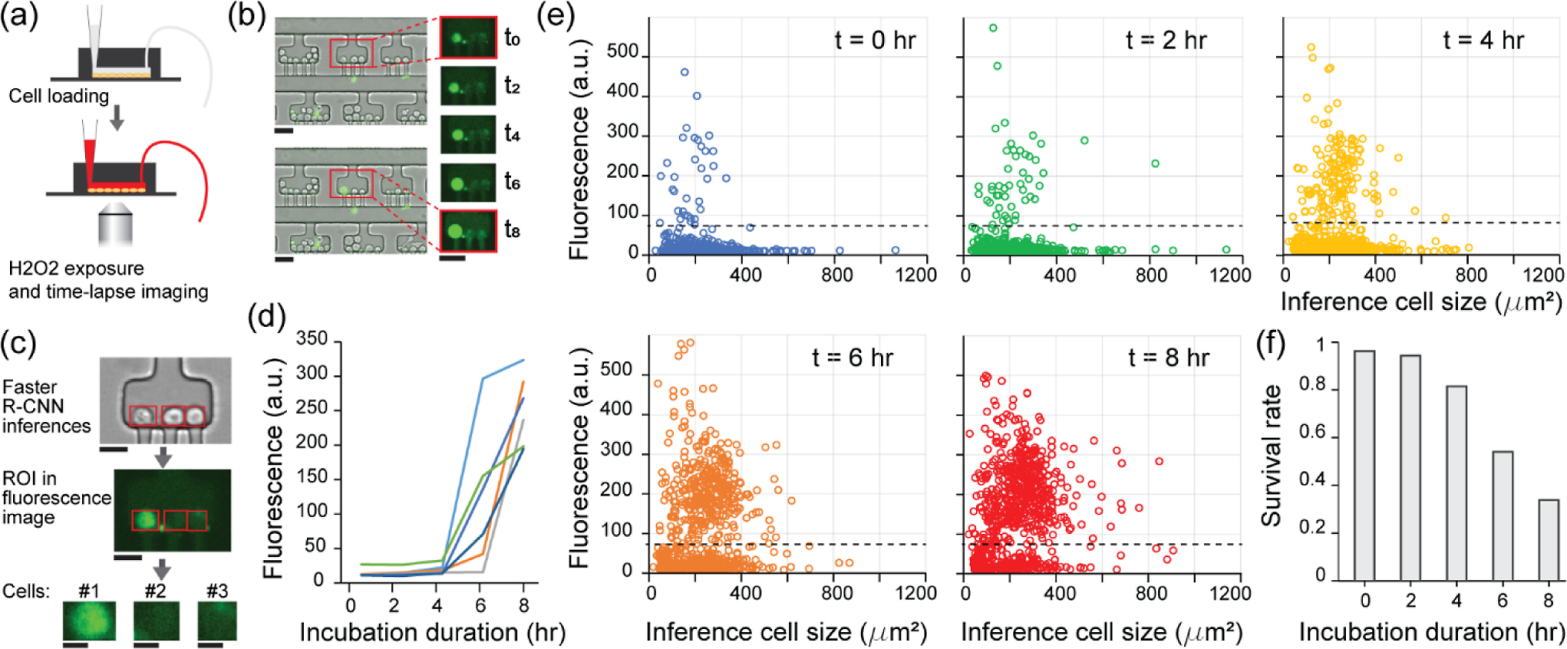
Application of Faster R-CNN cell detection in a simple killing assay. (a) Cell killing assay protocol: lymphoblast cells are loaded on chip. At t0, a 1 mM H_2_O_2_ solution is perfused. The cell array is then imaged every 2 h for 8 h. (b) Left: composite bright-field and fluorescence image of a microfluidic cell array at the beginning and the end of the killing assay (green: an apoptosis reporter). Right: time-lapse fluorescence images zoomed on one chamber. Scalebars: 40 μm. (c) Image analysis: bright field images are processed via the Faster R-CNN model. The resulting inferences are used in the fluorescence images to extract ROIs corresponding to individual cells. Scalebars: 20 μm for the images of the whole chamber and 10 μm for the fluorescence images of individual cells. (d) Longitudinal analysis of single cell responses. Each color indicates the average fluorescence intensity of an individual cell in a chamber. (e) Population response: each dot represents the mean fluorescence intensity of an individual cell. The dashed line represents the threshold of fluorescence chosen to distinguish healthy cells from dying cells. (f) Quantification of the fraction of cells alive during the assay.

To quantify the assay outcome, we used both the bright-field and fluorescence images (Figure 5c). First, we ran the Faster R-CNN model on the bright-field images to detect the cells. Next, we overlaid the bounding boxes generated by the model on the fluorescence images to quantify the amount of fluorescence corresponding to each cell. This allows to detect cells at fixed location in the chambers through the different time points and establish individual trajectories (Figure 5d).

To study the whole-population response, we represented the scattered plot of average fluorescence signal as a function of ROI size for all the cells on chip through time (Figure 5e). As time passes, a fraction of the data point cloud translates toward higher fluorescence values. The survival rate decreases slowly in the first 2 h and degrades to 30% by the end of the assay (Figure 5f). This trend is not surprising—that a larger fraction of the cell population enters apoptosis upon prolonged exposure to toxic chemical H_2_O_2_. Note that, because the control condition was imaged only at the beginning and end of the assay, the cells in the control condition received less light exposure than those in the treatment condition. Therefore, photoxicity might be a confounding factor of cell death in the treatment condition. Separate control experiments showed that K562 cells remained viable after repeated illumination over 15 h.

Using our trained Faster R-CNN model to perform the analysis took 4 h. The vast majority of that time corresponds to computer inferencing time without human supervision. This computing time can be drastically decreased using a GPU instead of a CPU. Still, manually analyzing the images or curating results from iLastik or MATLAB’s circle-detection method would take much longer, i.e. multiple days, and require human labor. These results highlight the benefit of a fully automated user-bias free method and its broad appeal to microfluidic cell assays.

Beyond killing assays, Faster R-CNN has broad potential in cell-based assays. More advanced phenotyping may be achieved using higher-content images. For example, at higher magnification, or with different microscopy modalities, it could enable identification of cell subtypes, detection of anomalous cells, or visualization of subcellular features such as organelles, vesicles, and synaptic boutons. Processing multispectral inputs, such as combinations of fluorescence channels and bright-field images, could further expand phenotyping capabilities. One should note that a limitation of Faster R-CNN is that it does not yield masks for features (e.g., cells or organelles) as its output, but instead bounding-boxes, which in turn provides limited morphological information, and thus may not be suitable for some assays. However, in cases where cell contours are needed, Faster R-CNN can still be valuable as a preprocessing step to isolate objects from the background in complex scenes. Because another key advantage of Faster R-CNN is its high computational speed, particularly with GPU acceleration, real-time detection could enable feedback-controlled on-chip applications such as flow cytometry or cell sorting. Altogether, Faster R-CNN offers versatile opportunities that can benefit a wide range of cell imaging applications.

## Conclusions

We have presented a facile image-processing method based on Faster R-CNN for cell detection in microfluidic assays. The neural network is pretrained on a broad image database, which reduces the training load with images of cells on chip. The annotation is easy and fast as the user draws boxes around the cells. A small amount images—as few as 40 cells—is required for training and for the resulting model to achieve high precision over 95%. In addition, we made available our pretrained model for detecting cells on chip, which can already achieve good accuracy without further modification. This model is a valuable tool to the microfluidic community.

Advanced assays may also benefit from this method. For example, Faster R-CNN based cell detection may be used in combination with fluorescence imaging or other modalities (e.g., quantitative phase imaging, or pathological staining). We demonstrated the use of the method for a killing assay that has a fluorescent apoptosis reporter. We showed one way to extract valuable single-cell resolution information from the fluorescence images using the cell inferences from bright-field images. This proof-of-concept may find extended benefits for other assays that combine fluorophores for cell tracking and gene expression reporters. We envision this method will enable the fast and easy processing of numerous cell-based assays in cell biology and the biomedical field.

## Supplementary Material





## Notes

Supporting Information A Faster R-CNN model trained to detect cell in microfluidic chambers (File S1) (PDF)
Detailed information on the images of the training set of the model used in Figure 2 (Figure S1). Comparison of inferences between a model trained on images of cells in wells and a model trained on images of cells in microfluidic chambers (Figure S2). Detection of HT180 adherent cells on chip (Figure S3). Screen captures showing representative results of CellPose-SAM processing images of cells in microfluidic chambers (Figure S4) (ZIP)
