# Supplementary material for: A Facile Method Based on Faster R‑CNN for Cell Detection in Microfluidic Devices
Source: Anal Chem. 2026 Jan 27;98(5):3557–65. doi: 10.1021/acs.analchem.5c04533 (PMC12903050; doi:10.1021/acs.analchem.5c04533)
Supplement: Supplementary file 1 [file ac5c04533_si_001.pdf]

Supporting Information for

A Facile Method Based on Faster R-CNN for Cell Detection in Microfluidic Devices

Guillaume Aubry,[a] Yanjun Zhao,[b] Erin Shappell,[c,d] Jacob M. Wheelock,[c,d] and Hang Lu [a*]

[a] School of Chemical & Biomolecular Engineering, Georgia Institute of Technology, 311 Ferst Drive NW, Atlanta, Georgia 30332, USA.

[b] College of Arts and Sciences, Troy University, Troy, Alabama 36082 USA.
[c] Interdisciplinary Program in Bioengineering, Georgia Institute of Technology, 311 Ferst Drive NW, Atlanta, Georgia 30332, USA.

[d] School of Electrical and Computer Engineering, Georgia Institute of Technology, 311 Ferst Drive NW, Atlanta, Georgia 30332, USA.

Corresponding Author
* Hang Lu: hang.lu@gatech.edu

Table of Contents

File S1: "Cell_detection_FasterRCNN_model.pth". Faster R-CNN model for detecting cells in a microfluidic chamber. The model was trained on 400 cell annotations and achieved an AP of 98 % on the testing set (see Figure 3a). This model was obtained using EZ-FRCNN package (https://ezfrcnn.com/) and can be run using the same package.

Figure S1: Detailed information on the images of the training set of the model used in Figure 2 ………………………………………………………………………….. p. S2

Figure S2: Comparison of inferences between a model trained on images of cells in wells and a model trained on images of cells in microfluidic chambers. ……….... p.S3

Figure S3: Detection of HT180 adherent cells on chip ……………..…………………. p.S4

Figure S4: Representative results of CellPose-SAM processing images of cells in microfluidic chambers ………………………………………………………………… p. S5

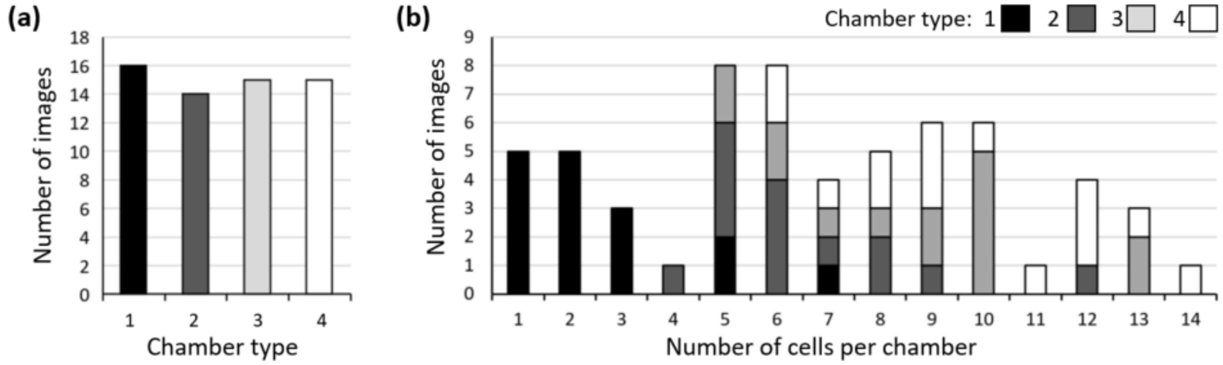

Figure S1. Detailed information on the images of the training set of the model used in Figure 2. There is an even distribution of images of each chamber type (a) covering a range of number of cells per chamber from 1 to 14 (b). The chamber dimensions are L x 40 µm x 15 µm where L = 50, 70, 90, and 110 µm for chamber types 1, 2, 3, and 4 respectively. The image distribution for different numbers of cells per chamber is relatively homogenous given the low number of images. There are an average of 6.8 cells per chamber and a standard deviation of 3.6 cells per chamber. Smaller chambers host lower number of cells. This is expected as the number of back channels is proportional to the chamber size: fewer cells in smaller chambers may lead to obstructing the back channels, blocking the transverse flow and stopping the hydrodynamic trapping effect.

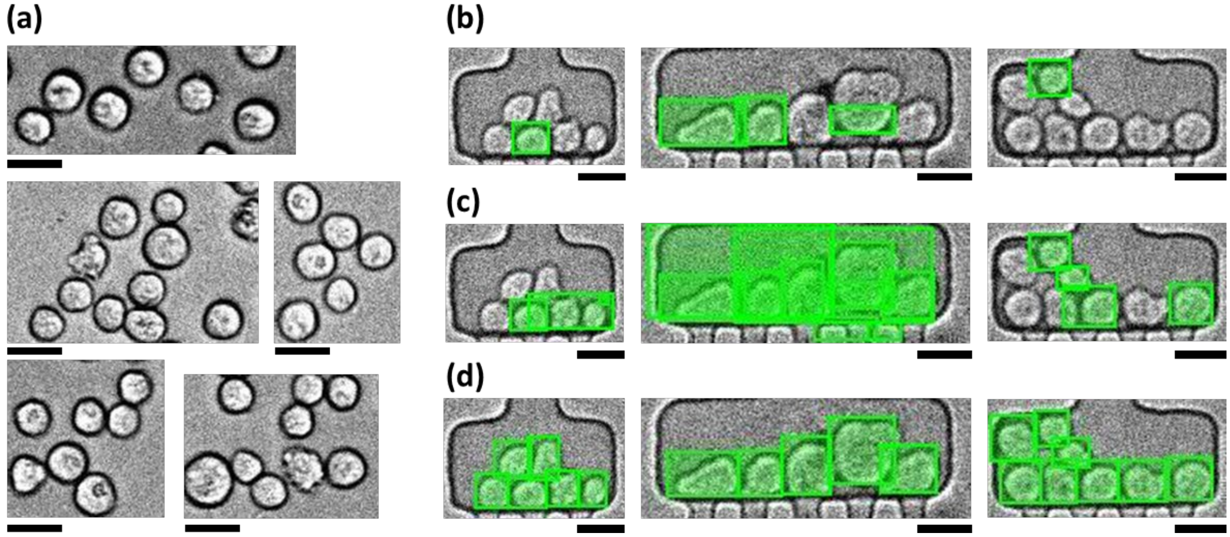

Figure S2. Comparison with a model trained on images of cells in wells. (a) Training set. (b) Representative images of the testing set with overlapped inferences generated by the model trained with images in (a) and for a confidence score threshold of 0.9. (c) Same images with overlapped inferences generated by the same model as in (b) and for a confidence score threshold of 0.1. (d) Same images with overlapped inferences generated by a model trained with 40 cell annotations of cells in microfluidic chambers. Scalebars are 20 μm.

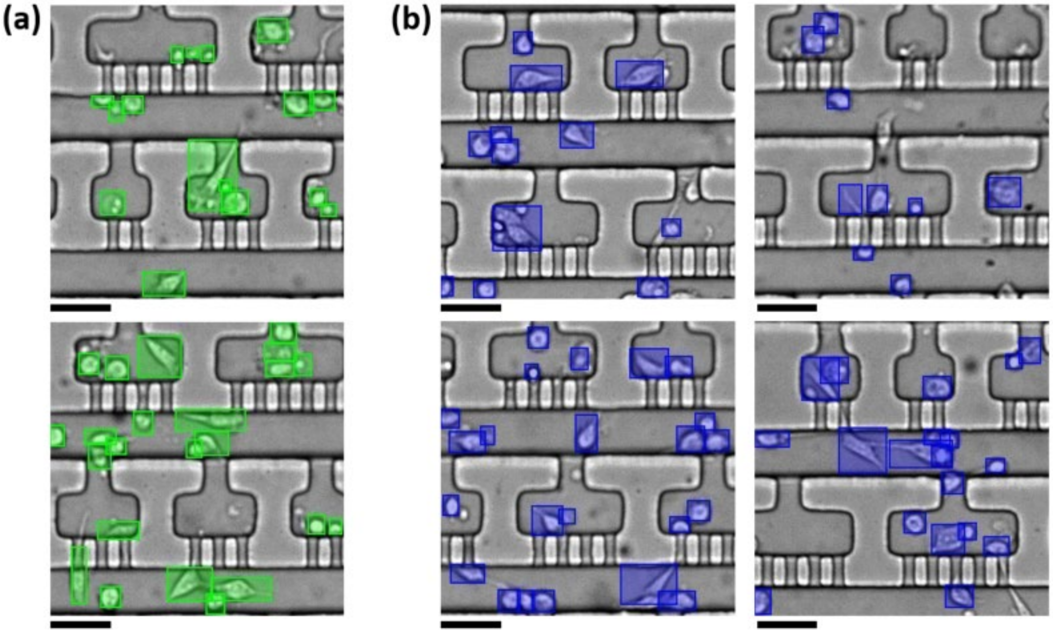

Figure S3. Detection of HT1080 adherent cells on chip. HT1080 fibrosarcoma cells were cultured in the same device as the K562 cells. The devices were previously coated with fibronectin to enable cell adhesion. A model was trained on two images containing 39 cell annotations and tested against 7 images containing 122 cells. (a) shows the training set with the annotations in green. (b) shows representative images of the testing set with the inferences overlapped in blue for a confidence score threshold of 0.8. Scalebars are 50 µm. Interestingly, when using the same parameters as for the detached cell analysis (*i.e.,* a confidence score threshold of 0.9 and IoU of 0.5), we obtain a precision of 91 % and a recall of 76 %. Lowering the parameter values to 0.8 and 0.3 for confidence score and IoU respectively leads to a precision of 91 % and a recall of 88 %. These results may be explained due to the larger variation in cell shape and intricate interaction of adherent cells with channel walls. Lowering the threshold of confidence score may be helpful for detecting cells whose shape diverge further from those used in the training set. Lowering the threshold of the IoU helps with scoring hits as inference and annotation boxes may differ strongly depending on how the cell protrusions are framed.

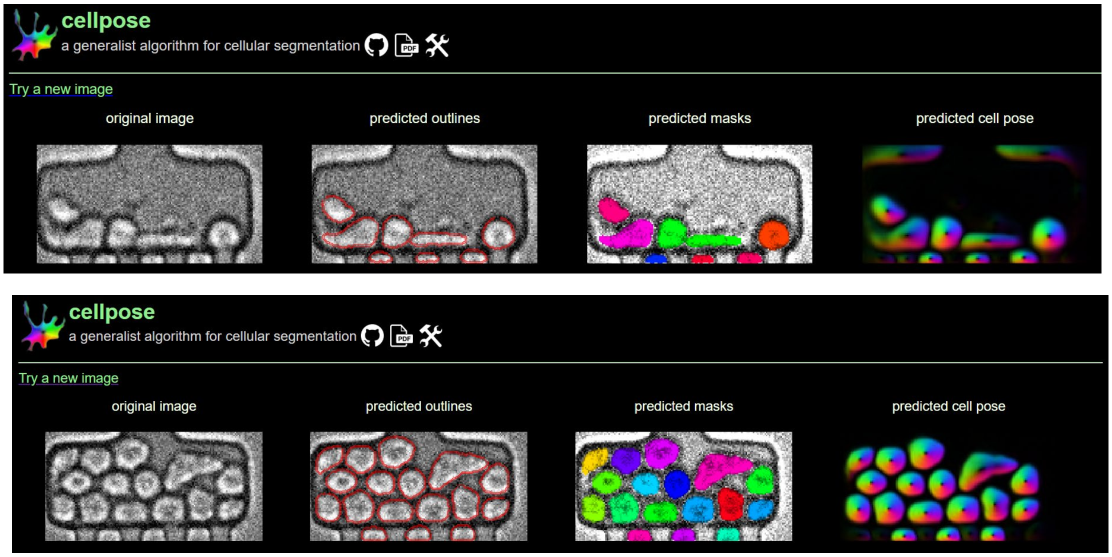

Figure S4. Screen captures showing representative results of CellPose-SAM processing images of cells in microfluidic chambers. One observes in the predicted outlines and predicted masks that multiple parts of the microfluidic channels are falsely detected as cells.
